# Integrated WiFi/PDR/Smartphone Using an Unscented Kalman Filter Algorithm for 3D Indoor Localization

**DOI:** 10.3390/s150924595

**Published:** 2015-09-23

**Authors:** Guoliang Chen, Xiaolin Meng, Yunjia Wang, Yanzhe Zhang, Peng Tian, Huachao Yang

**Affiliations:** 1 School of Environment Science and Spatial Informatics, China University of Mining and Technology, 1 Daxue Road, 221116 Xuzhou, China; E-Mails: chgl@cumt.edu.cn (G.C.); wyj4139@cumt.edu.cn (Y.W.); zyz@cumt.edu.cn (Y.Z.); tianp@cumt.edu.cn (P.T.); huachao.yang@cumt.edu.cn (H.Y.); 2 Nottingham Geospatial Institute, University of Nottingham, Triumph Road, NG7 2TU Nottingham, UK

**Keywords:** indoor localization, WiFi/PDR, clustering, auto-correlation analysis, Unscented Kalman Filter, Unity 3D

## Abstract

Because of the high calculation cost and poor performance of a traditional planar map when dealing with complicated indoor geographic information, a WiFi fingerprint indoor positioning system cannot be widely employed on a smartphone platform. By making full use of the hardware sensors embedded in the smartphone, this study proposes an integrated approach to a three-dimensional (3D) indoor positioning system. First, an improved K-means clustering method is adopted to reduce the fingerprint database retrieval time and enhance positioning efficiency. Next, with the mobile phone’s acceleration sensor, a new step counting method based on auto-correlation analysis is proposed to achieve cell phone inertial navigation positioning. Furthermore, the integration of WiFi positioning with Pedestrian Dead Reckoning (PDR) obtains higher positional accuracy with the help of the Unscented Kalman Filter algorithm. Finally, a hybrid 3D positioning system based on Unity 3D, which can carry out real-time positioning for targets in 3D scenes, is designed for the fluent operation of mobile terminals.

## 1. Introduction

Recently, a variety of indoor positioning technologies have emerged, such as ultrasound [1], infrared [2], Wireless Local Area Network, Bluetooth, Radio Frequency Identification [3], and Ultra Wideband. The RADAR [4], LANDMARC, and Place Lab techniques are the most representative within those approaches. However, these systems require additional hardware devices and complicated deployment. With the development of intelligent mobile terminals (such as smartphones and tablet computers), these devices can provide additional unprecedented functions. In addition, they can also provide a number of advanced technologies, such as WiFi, Bluetooth, and inertial sensors. A developer can make full use of these technologies and hardware to invent an indoor positioning system that is much easier to operate and less costly.

WiFi fingerprint indoor positioning systems focus on improving positioning accuracy and real-time performance [5,6,7]. There are two main methods to improve positioning accuracy, improving the positioning algorithm [8] or increasing the density of the fingerprint [9]. However, increasing the density of the fingerprint reduces the real-time performance because more fingerprint matching time is required whether based on direct interpolation positioning [10] or probability distribution positioning [11]. Especially when running on a mobile terminal, the time consumed by WiFi fingerprint positioning on limited hardware cannot meet real-time requirements. Therefore, improving the algorithm by lessening the amount of calculation is key to improving the effectiveness and positioning accuracy. Clustering the fingerprint database [12] is a good way to control the scale of the sample point search. The employment of a classical K-means clustering algorithm greatly assists the sub block processing of the WiFi fingerprint. With clustering, the time consumed by positioning is sharply decreased while the system precision is ensured, which effectively improves the real-time performance.

Pedestrian Dead Reckoning (PDR) positioning along with the use of the Inertial Measurement Unit that is built into a smartphone is widely used in navigation applications in indoor environments. Using PDR, a pedestrian’s next position can be calculated when the starting position, heading information, and displacement are known. Many researchers use a step length model combined with direction information [13,14] to calculate the displacement of indoor pedestrians. However, the peak detection algorithm [15,16], which is commonly used for counting steps, cannot work well because of the large error of a smartphone. A new step counting method based on auto-correlation analysis of the sensor data is explored here. Compared with the traditional peak detection algorithm, it can clearly reduce the influence of step counting result errors caused by different mobile phone locations and motion postures of pedestrians.

PDR can provide motion information at a high update rate and achieve high precision over a short time duration. However, without external aids, the system suffers from local anomalies and cumulative error after positioning for a longer length of time. The positioning accuracy of WiFi fingerprinting depends on the density of the training samples, which means it does not have an accumulative error problem. Given the complementary characteristics of these two methods, combination of the two sources would bring better performance than a single source. Leppakoski [17] proposes Complementary Extented Kalman Filter (CEKF) for the fusion of PDR and WiFi positioning. Anshul [18] uses augmented particle filtering to simultaneously estimate location and user-specific walk characteristics, while the algorithm occupy large source and in open areas particle is less effective. Since the whole system is running on a resource limited smartphone, Chen zhenghua [19] formulates sensor fusion problem in a linear perspective and apply Kalman filter instead. However, the linearization of model is limited, the author attempts to integrate them with Unscented Kalman Filtering (UKF) algorithm. UKF is a nonlinear filtering algorithm proposed by Julier and based on the Unscented (U) transform. It can overcome the limitations of lower filter precision-caused truncation error and the necessity for a Jacobian matrix, and has been widely applied in nonlinear estimation [20].

With the development of the indoor positioning technology, there are a few of apps in initial stage. Xunlu is the first indoor navigation application for airports and markets in China which is based on WiFi positioning. However, it is easily influenced by the environment such as crowds which need multiple WiFi routers and adjust the relationship of WiFi signal intensity and location. Ibeacon is for IOS(iphone Operation System) based on BLE (Bluetooth Low Energy) location technology. Besides, IndoorAtlas developed an IndoorAtlas Application utilizing geomagnetic map, rather than WiFi or BLE. All these apps use single indoor positioning technology while no integration of different location methods. Most indoor positioning systems currently provide location services based on two-dimensional (2D) maps. The three-dimensional (3D) navigation and location system first appeared in the 2010 World Exposition in Shanghai [21]. Traditional 2D map is the abstraction of the indoor geographic information and cannot effectively express indoor geographic information because of the complexity of indoor environments, so it makes those users who are not professional difficult to identify his location. While 3D map express the actual three-dimensional space of indoor scene intuitively and realistic and show details of complex indoor environment, which could help people quickly figure out where he is and the direction he face. Moreover, smartphones cannot process the large amount of calculation needed for three-dimensional (3D) visualization. As a result, 3D visualization in mobile terminals has become an urgent problem to be solved. Mobile terminal 3D visualization relies on the Unity 3D platform, which enhances the expressiveness of indoor geographic information and user experience. The goal of this study is to design and develop a 3D indoor positioning system that locates indoor targets using a mobile terminal and monitors these indoor targets in a 3D scene.

The rest of this paper is organized as follows: Section 2 introduces WiFi fingerprint positioning and clustering of fingerprint databases. Section 3 presents an experimental analysis of PDR with a new step counting method. Section 4 presents the fusion of WiFi fingerprint positioning and PDR positioning using the UKF algorithm to improve accuracy. Section 5 shows how to achieve the 3D visualization in a mobile terminal using Unity 3D. Section 6 concludes and discusses the study’s contributions.

## 2. WiFi Fingerprinting Positioning

**Figure 1:**
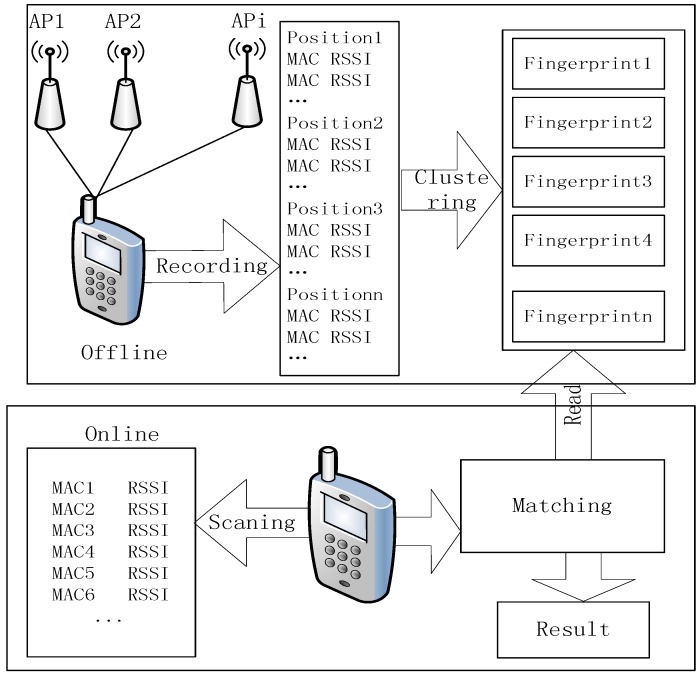
Positioning process.

A typical approach of WiFi fingerprint positioning consists of an offline phase and online phase. The offline phase is the first step to obtaining several access points (APs) that maintain a certain distance from each other in the positioning area. The data of each AP forms the sample point sets, denoted as *L* = {*l*_1_, *l*_2_, …, *l_m_*}. Each element in *L* consists of two parts: *Pi* = (*xi*, *yi*) denotes the coordinates of each location and *Vi* = [*vi*, *1*, *vi*, *2*, …, *vi*, *n*] is the Received Signal Strength (RSS) vector received from the APs at the *i*-th reference point. The final database is generated by clustering using the K-means algorithm. In the online phase, the tracking target collects real-time RSS from APs and matches them with the fingerprints stored in the database to obtain the final position. The overall positioning process is shown in Figure 1.

### 2.1. WiFi Fingerprint Clustering

The primary clustering algorithms at present are the partitioning, hierarchical, density-based, grid-based, and model-based methods. The most typical method is the K-means clustering algorithm, which is a partitioning-type clustering method. Given the criterion for classification and the number of clusters K, this algorithm can divide a database of n data objects into K parts. The data within one cluster are extremely similar but are clearly different to those of other clusters.

WiFi fingerprint clustering divides sample points into several groups based on the signal distance between each two sample points. The distance is calculated as follows:

(1) $$SD(l_{i},l_{j})=\|v_{i},v_{j}\|=\sum_{k=1}^{n}\sqrt{{\left(v_{i,k}-v_{j,k}\right)}^{2}}$$

The K-means algorithm clusters the fingerprint database as follows: First, k points are randomly picked as the initial center for each cluster. According to the distance from each center of the other clusters, the rest of the sample points are attached to the nearest cluster. The average value of each cluster is then recalculated. This recalculation repeats until the distance between each sample point and its clustering center are minimized. The calculation of E is as follows:

(2) $$E=\sum_{i=1}^{k}\sum_{l\in C_{i}}\left\|l-c_{i}\right\|$$

where *c_i_* is the average value of cluster *C_i_*.

### 2.2. WiFi Fingerprint Positioning Algorithm

The traditional algorithm for WiFi fingerprint positioning is K Nearest Neighbor in Signal Space (K-NNSS), proposed by Bahl [4]. When positioning begins, real-time RSS measurements from the APs are collected to form vector *SV* = [*v*_1_, *v*_2_, …, *v_n_*], *v_i_*(1 ≤ *i* ≤ *n*). The similarity *Length_i_* to each sample point in the fingerprint database is calculated as follows:

(3) $$Length_{i}=\left\|SV,v_{i}\right\|=\sum_{k=1}^{n}\sqrt{{\left(v_{k}-v_{i,k}\right)}^{2}}$$

All values of *Length* are ranked in ascending order and the *k* minimum *Length* coordinates are selected to form a vector *RL* = {*RL*_1_, *RL*_2_, …, *RL_k_*}, (1 ≤ *i* ≤ *k*, *RL_i_* = (*x_i_*, *y_i_*)). The final positioning result is as follows:

(4) $$RL=\sum_{j=1}^{k}w_{j}{RL}_{j}$$

where *w_j_* is the weight of coordinate *j*.

The location area is too large for a sampling point to receive all AP signals, so APs which different sampling points in the fingerprint database can receive are different. The number of the same APs two sampling points can received signals from is larger, the two sampling points is closer. Therefore, the number of the same APs described above reflects spatial distance relationships of the two sampling points. Hence, taking the factor of spatial distance into the similarity calculation, the number of the same APs is utilized to improve similarity shown as below:

(5) $${Length}_{i}=(1-\frac{{NUM}_{s}}{NUM})\sum_{k=1}^{n}\sqrt{{\left(v_{k}-v_{i,k}\right)}^{2}}$$

Here, *NUM* is the number of APs in the sampling area, and *NUM_s_* is the number of these APs that can be sensed at the sampling point at the same time.

### 2.3. Experiment and Results

#### 2.3.1. Experimental Setup

The corridor of the fourth floor corridor in the School of Environment Science and Spatial Informatics, China University of Mining and Technology was chosen as the test area. The building’s general WiFi signal was used as the ubiquitous signal for positioning. There were 216 available APs in this 670 m^2^ area.

**Figure 2:**
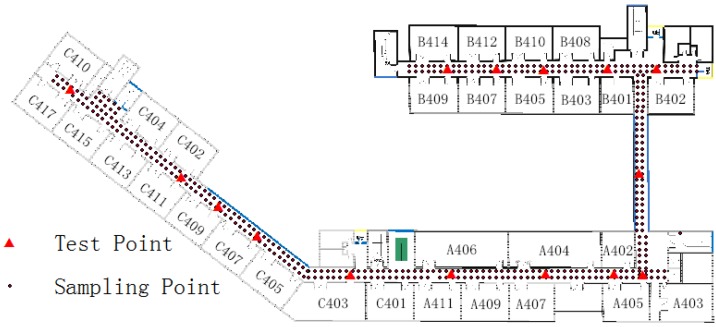
Distribution of sampling and test points.

This experiment consisted of a sampling phase, offline phase, and positioning phase. A Samsung Galaxy S III (Android 4.0) (Samsung Electronics, Seoul, Korea) was used as the device for sampling and positioning, and the sampling rate was 1 Hz. The RSS value received from each AP was an integer between −110 and 15. Each sampling point continuously offered 25 samples, and researchers recorded their average signal strength. In the offline phase, 390 available sampling points were collected (the average sample interval was 3.5 m) and stored in a database as fingerprints, which were processed using the K-means clustering algorithm. In the online positioning phase, 15 points were randomly chosen as test points and each were located 10 times to verify the algorithm’s accuracy. The distribution of the sampling and test points are shown in Figure 2:

#### 2.3.2. Clustering Test

**Figure 3:**
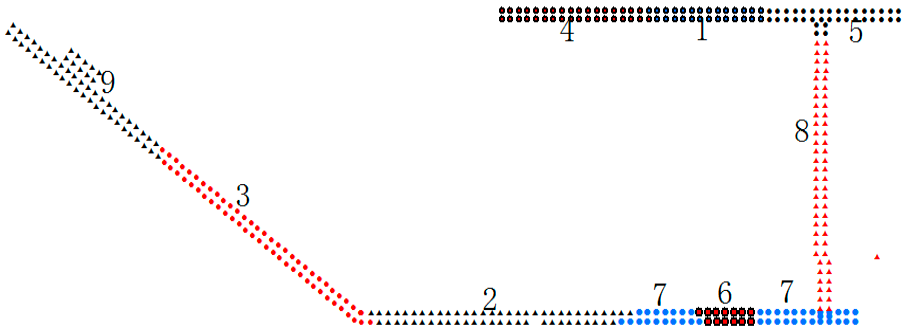
Clustering results.

Three hundred and ninety sampling points were marked in sequence and configured as a data matrix (390 × 212) using Equation (3). The K-means algorithm is a supervised learning method and needs to be given the cluster number and initial center point. In this test, the cluster number was nine and the initial center was randomly picked. The clustering results are shown in Figure 3:

The number of sampling points in each cluster is listed in Table 1.

**Table 1:** Number of sampling points in each cluster.

**Class Number**	1	2	3	4	5	6	7	8	9
**Point Number**	27	58	62	35	36	13	43	61	55

According to the results, the indoor environment clearly affects the clustering result, and the boundaries of different indoor environments are relevant to the boundaries of the clusters.

#### 2.3.3. Positioning Test

The K-NNSS algorithm was used for positioning, where K was set to four. Considering that the number of repeated APs can reflect the spatial relationship, the improved similarity calculation in Equation (5) was selected to calculate *Length*. The Samsung Galaxy Ⅲ (Android 4.0) was also chosen for the positioning test. Fifteen randomly selected test points were continuously located 10 times. A comparison between clustered WiFi fingerprints and unclustered WiFi fingerprint was made with respect to three factors: average positioning time, average error, and maximum error.

**Figure 4:**
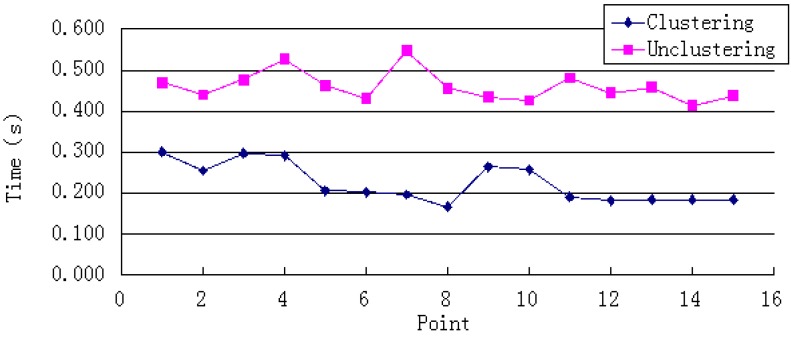
Single-point positioning time.

Clustered WiFi fingerprints can reduce the positioning time by reducing the amount of data retrieved. As Figure 4 shows, the system’s positioning time sharply decreases; the average reduction is 51%, the maximum reduction is 64%, and the minimum reduction is 36%.

**Figure 5:**
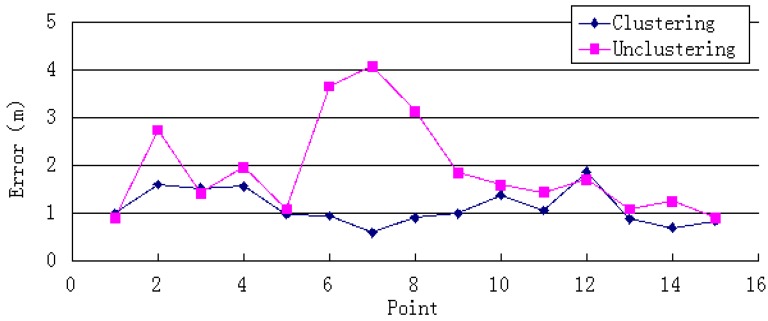
Single-point positioning average error.

**Figure 6:**
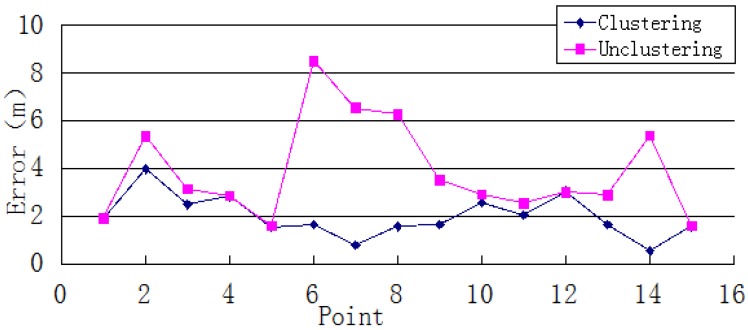
Single-point positioning maximum error.

As Figure 5 and Figure 6 show, the system’s average and maximum errors indicate that the single-point positioning results are more accurate after clustering. The average error was decreased from 1.92 to 1.12 m. The largest improvement was a reduction from 4.09 to 0.59 m. The average maximum error of single-point positioning decreased from 3.87 to 2.00 m, and the maximum reduction was 6.85 m. These results imply that the clustering algorithm can narrow the scope of the position search and reduce the error of the positioning result caused by instability of the WiFi signal.

## 3. Inertial Positioning

**Figure 7:**
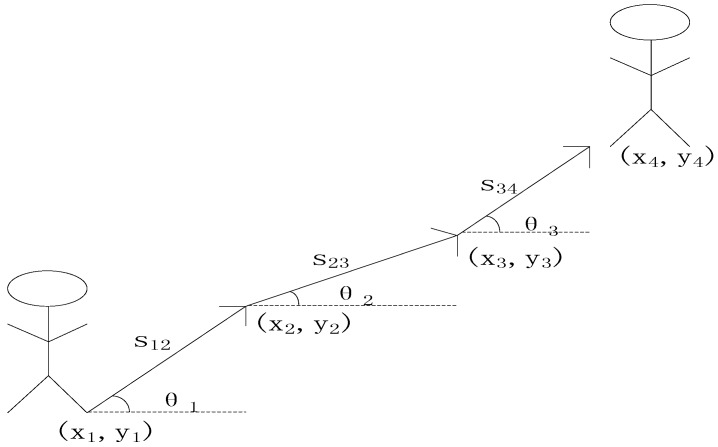
PDR principle.

Typically, a pedestrians’ movement over a very short sampling time is regarded as rectilinear motion In PDR, a pedestrian’s next position can be deduced from the starting position, heading information, and displacement. The principle of PDR is shown in Figure 7:

If the initial position is assumed to be (*x_i_*, *y_i_*), the calculation of the next position (*x*_2_, *y*_2_) is:

(6) $$y_{2}=y_{1}+S_{12}\cdot \text{cos}\theta_{1}$$

(7) $$x_{2}=x_{1}+S_{12}\cdot \text{sin}\theta_{1}$$

Equations (6) and (7) can be easily adapted to obtain the next step’s position. There are two key factors during the whole calculation: displacement *s* and heading direction *θ*. Of these, *s* can be estimated using a typical frequency-step model and *θ* can be obtained using an orientation sensor or gyroscope.

### 3.1. Counting Steps

**Figure 8:**
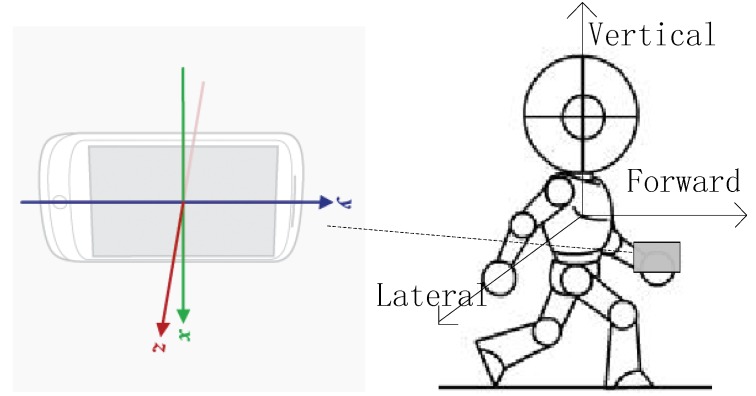
Schematic of coordinate axes.

The body’s natural walking motion includes three components: forward, lateral, and vertical. These components with respect to a mobile phone’s coordinate axes are shown in Figure 8.

**Figure 9:**
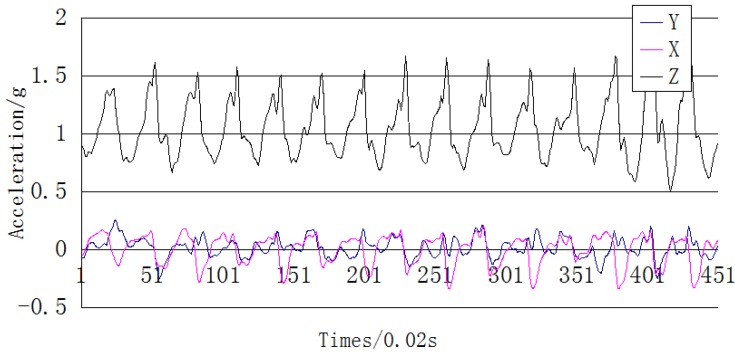
Three-axis acceleration of a mobile phone.

The vertical acceleration in a step cycle changes regularly along with foot up-and-down movements. The acceleration changes along the three axes are shown in Figure 9. According to the clear periodicity of the *z*-axis acceleration, a step-counting algorithm based on the auto-correlation analysis of acceleration is proposed that can greatly reduce the error of step counting results caused by different orientations of the mobile phone and individual motion states.

#### 3.1.1. Auto-Correlation Analysis

The proposed auto-correlation analysis algorithm counts steps according to the similar acceleration of a pedestrian continuous motion. It divides the motion state into two cases: idle and walking. Stationary status includes the static state, standing up, sitting down, turning, gesturing, and other actions that do not change the body’s position. The walking status occurs when the body changes its position while the mobile phone is being carried or used. There are two steps to calculating each step cycle: calculation of the standard deviation and calculation of the auto-correlation.

(1) Calculation of Standard Deviation

To reduce the influence of the phone’s orientation, the overall acceleration is chosen to count steps. The overall acceleration described as follows:

(8) $$a=\sqrt{{a_{x}}^{2}+{a_{y}}^{2}+{a_{z}}^{2}}$$

where *a_x_*, *a_y_* and *a_z_* are the acceleration of the three axes. We identify the motion state using the standard deviation *σ* of *a*:

(9) $$\sigma =\sqrt{\frac{\sum_{k=1}^{k=N}{\left(a_{k}-u\right)}^{2}}{N}}$$

where u is the mean of {*a*_1_, *a*_2_, …, *a_n_*}.

**Figure 10:**
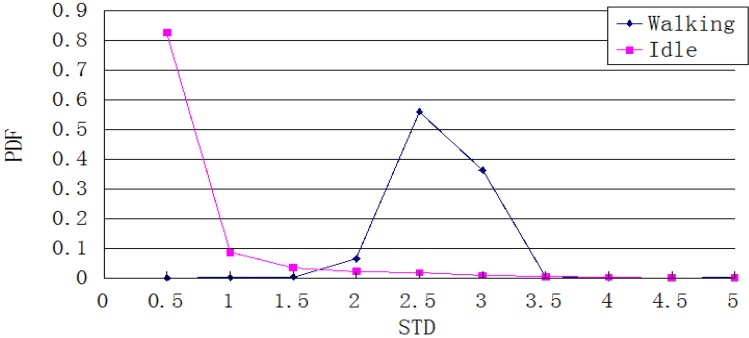
Distribution of the standard deviation of the overall acceleration in stationary and walking states.

Figure 10 shows the Probability Density Function of the overall distribution of the acceleration standard deviation during a 1-s cycle, after recording walking and stationary statuses each for 5000 times. When the standard deviation is under 0.5, the probability that the pedestrian is stationary is greater than 99%. This statistic can be used as a heuristic threshold to judge the pedestrian’s state, e.g., the pedestrian is considered to be stationary when the standard deviation is under 0.5. However, this value is not accurate enough to be used as a threshold for other states such as turning or some basic gestures. These motions have a larger standard deviation. To deal with this situation, an auto-correlation calculation is a better method to use.

(2) Calculation of Auto-correlation

**Figure 11:**
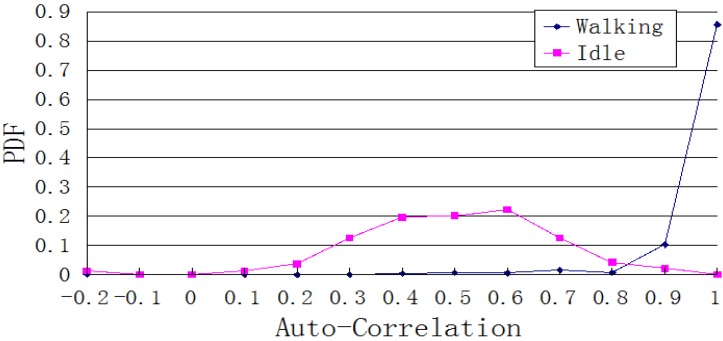
Distribution of auto-correlation during stationary and walking states.

According to Figure 11, the acceleration periodically changes because of the pedestrian’s cyclical step movements. This characteristic can be applied to determine the auto-correlation between the current and previous strides, and hence identify the walking state.

The mobile phone acceleration sensor continuously records data when the pedestrian is walking. The overall auto-correlation of the acceleration is determined as follows:

(10) $$x(m,t)=\frac{\sum_{k=0}^{k=t-1}\left[(a(m+k)-u(m,t))\times (a(m+k+t)-u(m+t,t))\right]}{t\times \sigma (m,t)\times \sigma (m+t,t)}$$

where *u*(*m*, *t*) and *σ*(*m*, *t*) are the mean and standard deviation of the sequence samples {*a*(*k* + 1), …, *a*(*k* + *t* + 1)}. When the sampling period is close to the stride period, *x*(*m*, *t*) is approximately one. However, different individuals or one individual at different times can have different stride frequencies, so *t* is a varied. The frame algorithm is designed to obtain *t* dynamically. The algorithm selects *t* from the range *t*_min_ − *t*_max_, then calculates *ρ*(*m*, *t*) as follows:

(11) $$\rho (m,t)={\text{max}}_{t=t_{\text{min}}}^{t=t_{\text{max}}}(x(m,t))$$

where *t* is the period of the acceleration pattern that *ρ*(*m*, *t*) is maximized.

The stride frequency of normal walking ranges from 1 to 2.5 Hz, so *t* ranges from 0.4 to 1 s. Figure 11 presents the Probability Density Function of *ρ*(*m*, *t*) for both stationary and walking state, each sampled 1000 times. Based on Figure 11, when *ρ*(*m*, *t*) is 0.7 or higher, the probability of a walking state is greater than 90%.

#### 3.1.2. Experiment and Results

According to the two thresholds for judging the motion state, auto-correlation algorithm is carried out. Setting *t* to 0.4−1.0 s and calculating the standard deviation of the overall sequence, the motion state was determined as follows:

(1) If *σ* < 0.5 then the state is stationary.
(2) If *ρ*(*m*, *t*) > 0.7 then the state is walking.
(3) Otherwise, the current state is unchanged.

Table 2 lists the test results for the auto-correlation algorithm and the peak detection algorithm.

**Table 2:** Performance of the auto-correlation algorithm.

Position of Mobile Phone	Motion State of Pedestrian	Peak Detection Algorithm Steps	Auto-Correlation Algorithm Steps	True steps
In Hand	Changing frequency of walking	224	196	200
In Jacket pocket	Constant speed walking	233	202	200
In Hand	phone using when walking	204	198	200
In Hand	Constant speed walking	199	197	200
In Hand	Idle	0	0	0
Change positions	Idle	6	6	0

According to the chart, the auto-correlation algorithm greatly reduces the influence of different mobile phone positions and various pedestrian motions compared to the peak detection algorithm. It can hence be applied to the step counting task in all situations.

### 3.2. Step Length Estimation

A frequency model is chosen as the generic step model calculated as follows:

(12) $$L_{g}=a\times f+b$$

where *f* is walking frequency, and *a* and *b* are coefficients. The step length is difficult to set because of the different height and weight of individuals. The coefficient values are adopted from Li, who obtained parameters from 4000 steps of 23 different people [22].

### 3.3. Heading Direction Estimation

Heading direction can be determined by compass or gyroscope. Magnetic direction can be directly obtained from a direction sensor (compass), which is easily disturbed by circumstances. The magnetic heading angle is calculated as:

(13) $$h_{mag}=\text{arctan}(\frac{m_{y}}{m_{x}})$$

where *m_x_* and *m_y_* are the Earth’s magnetic field components along *x* and *y* axes of the local frame.

The strapdown heading direction comes from the gyroscope angular velocity and initial direction. It is stable when faced with external disturbance, but accumulates more error. The strapdown heading direction can be determined by integrating the *z*-axis angular velocity of the gyroscope:

(14) $$h_{k}=h_{k-1}+w_{k}dt$$

where *h_k_* is current strapdown heading direction and *w_k_* is the angular velocity at step *k*. To reduce the circumstance disturbances as well as accumulative error, this study adopts a combination of both approaches to determine the heading direction, calculated as follows:

(15) $$a=(1-W)h_{k}+{Wh}_{mag}$$

where *W* is the weight of the magnetic data. The value of *w* is taken from [23].

However, obvious error could exist in the data from the mobile phone sensors, or the phone might not be oriented along the motion direction of the user. Given this situation, this study uses map information to constrain the heading direction.

## 4. UKF

PDR has a high precision over a short time, but gradually accumulates error. In contrast, WiFi fingerprint positioning has no accumulative error. The combination of WiFi fingerprint and PDR positioning leads to a more stable indoor positioning over the long term.

Error in PDR is mainly caused by the random error of various sensors (accelerometer, gyroscope, and compass). Hence, the system’s positioning accuracy and reliability can be improved effectively using an optimal estimation method. The Extended Kalman Filter (EKF) is a simple and popular formulation for nonlinear estimation [24,25,26]. However, it causes model error in the linearization and reduces estimation accuracy. In addition, a high-dimensional complicated model called the Jacobi Matrix must be calculated for the EKF. In the 1990s, Julier proposed the UKF method, which uses the U transform in the filtering process to improve accuracy.

### 4.1. UKF Model

Assume a system dynamic model and measurement model as follows:

(16) $$\begin{array}{l} X_{k}=f(X_{k-1},u_{k-1})+W_{k-1}\\ Z_{k}=h(X_{k})+V_{k} \end{array}$$

where *f* and *h* are the nonlinear vector functions, *W_k_* and *V_k_* are the process noise and measurement noise, respectively, which are both uncorrelated zero mean white Gaussian noise, and their covariances are *Q_k_* and *R_k_*, respectively. Finally, *u*_*k*−1_ is the control input of the model.

A UKF is a Kalman Filter based on the U transform. Suppose an N-dimensional random variable $X\sim N(\bar{X},P_{X})$. Further, Z is the statistic characteristic $\left(\bar{Z},P_{Z}\right)$ transformed from X according to a nonlinear function *f*(⋅). The U transform designs a series of points *ξ_i_*(*i* = 1, 2, …, *L*) named Sigma points using $\left(\bar{X},P_{X}\right)$, and calculates the result *χ_i_*(*i* = 1, 2, …, *L*) of the Sigma points using *f*(⋅) to obtain the result $\left(\bar{Z},P_{Z}\right)$ based on *χ_i_*. Generally, the number of Sigma points is 2n + 1. The U transform can be described as follows:

#### (1) Calculation of Sigma points

(17) $$\left\{ \begin{array}{l} \xi_{0}=\bar{X}\\ \xi_{i}=\bar{X}-{\left(\sqrt{(n+\lambda )P_{X}}\right)}_{i},(i=n+1,n+2,\cdots ,2n)\\ \xi_{i}=\bar{X}-{\left(\sqrt{(n+\lambda )P_{X}}\right)}_{i},(i=n+1,n+2,\cdots ,2n) \end{array}\right.$$

(18) $$\left\{ \begin{array}{l} \omega_{0}^{m}=\frac{\lambda }{n+\lambda }\\ \omega_{0}^{c}=\frac{\lambda }{n+\lambda }+(1-\alpha^{2}+\beta )\\ \omega_{i}^{m}=\omega_{i}^{c}=\frac{1}{2(n+\lambda )},i=1,2,\cdots ,2n \end{array}\right.$$

(19) $$\lambda =\alpha^{2}(n+k)-n$$

where *a* is usually a small positive number such as 0.001, *k* = 0, *β* is usually used to describe the information distribution of *X* (the optimal value of *β* is two in a Gaussian noise environment). Further, ${\left(\sqrt{(n+\lambda )P_{X}}\right)}_{i}$ represents column *i* of the square root matrix, and $\omega_{i}^{m}$ and $\omega_{i}^{c}$ are the weight coefficients for calculating first- and second-order statistical characteristics.

#### (2) Calculation of *χ_i_*, *P_ZZ_* and *P_XZ_*

(20) $$\chi_{i}=f(\xi_{i}),i=0,1,\cdots ,2n$$

(21) $$\left\{ \begin{array}{l} \bar{Z}=\sum_{i=0}^{2n}\omega_{i}^{m}\chi_{i}\\ P_{ZZ}=\sum_{i=0}^{2n}\omega_{i}^{c}(\chi_{i}-\bar{Z}){\left(\chi_{i}-\bar{Z}\right)}^{T}\\ P_{XZ}=\sum_{i=0}^{2n}\omega_{i}^{c}(\xi_{i}-\bar{Z}){\left(\chi_{i}-\bar{Z}\right)}^{T} \end{array}\right.$$

**Figure 12:**
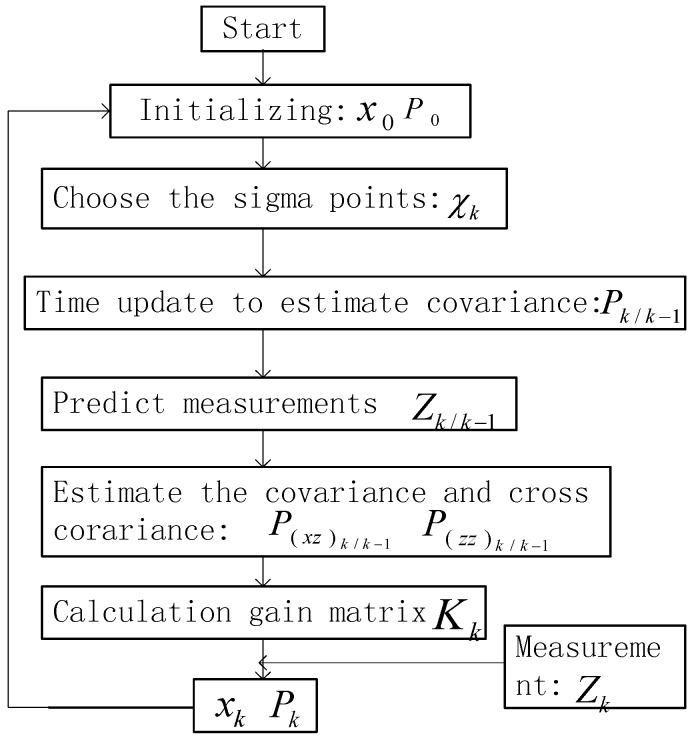
UKF process.

A flow chart of the UKF is presented in Figure 12.

### 4.2. Implementation of UKF Fusion

UKF is used to fuse the results of WiFi fingerprint positioning and PDR positioning. The UKF fusion is achieved by directly using step domain instead of time domain, which could eliminate the process of converting the PDR location to the distance meter before fusion with WiFi positioning information, and reduce the complexity of the algorithm. According to the pedestrian state, a system model is built with the nonlinear formula as follows:

(22) $$X_{k}=\left(\begin{array}{c} x_{k}\\ y_{k}\\ \theta_{k} \end{array}\right)=\left(\begin{array}{c} x_{k-1}+\tilde{s}\cdot \text{cos}\theta_{k-1}\\ y_{k-1}+\tilde{s}\cdot \text{sin}\theta_{k-1}\\ \theta_{k-1}+\tilde{\theta } \end{array}\right)+W_{k-1}$$

where *x_k_* and *y_k_* are the position after *k* steps, *θ_k_* is the heading direction after *k* steps,*W*_*k*−1_ is three-dimensional system process noise, $\tilde{s}$ is the step length of step *k*, and $\tilde{\theta }$ is the variation of the heading direction in step *k*. The measurement model is created as follows:

(23) $$Z_{k}=\left(\begin{array}{c} x_{k}\\ y_{k}\\ s_{k}\\ \Delta \theta_{k}\\ \theta_{k} \end{array}\right)=\left(\begin{array}{c} x_{k}\\ y_{k}\\ \sqrt{{\left(x_{k}-x_{k-1}\right)}^{2}+{\left(y_{k}-y_{k-1}\right)}^{2}}\\ \theta_{k}-\theta_{k-1}\\ \theta_{k} \end{array}\right)+V_{k}$$

where *x_k_* and *y_k_* are the position of pedestrian from WiFi fingerprint positioning, *s_k_* is length of step *k* from PDR positioning, △*θ_k_* is the variation of the heading direction of step *k* from the gyroscope, *θ_k_* is the heading direction of step *k* from the compass, and *V_k_* is five-dimensional system measurement noise.

### 4.3. Experiment and Results

**Figure 13:**
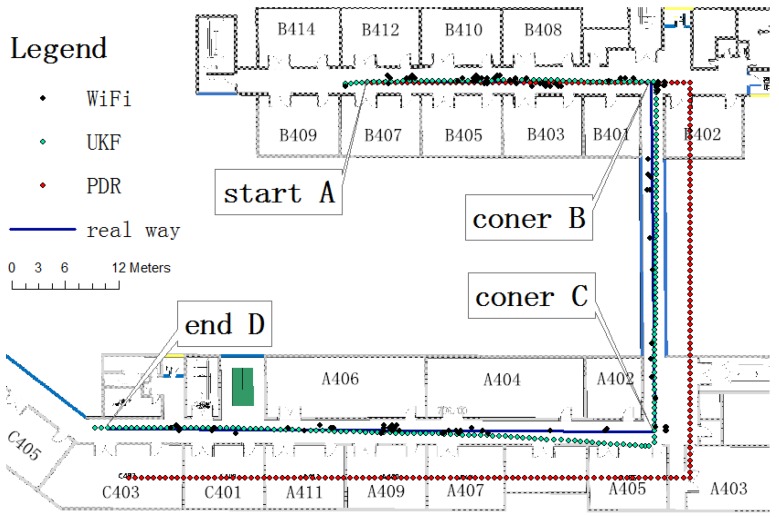
UKF positioning test.

The location experiment was carried out in zones A and B on the fourth floor of the School of Environment Science and Spatial Informatics, China University of Mining and Technology. The initial location of the pedestrian subject was known. The tester moved from the starting point shown in Figure 13 to the end after two right-angle corners, for a total of 133.5 m. The initial positioning performance coincided with the real path, but declined over time. According to Figure 13, the accumulative error reached 3.5 m at corner B, and reached 6.8 m at corner C. The WiFi fingerprint positioning has a large error and, in some cases, its position result concentrated on a small area. As the WiFi fingerprint positioning result was used for the filter initial value, the initial position error was obvious, about 2.3 m in corner A. As time went by, the fusion result lessened the error, which was just under 1 m in corner B and 1.7 m in corner C. Compared with the 6.8-m error of PDR positioning only, the error was greatly improved. When the tester reached end D after 133.5 m, the PDR positioning error was 6.0 m. In contrast, the fusion positioning error was just 1.5 m. From this experiment, it can be concluded that the UKF filter algorithm could fuse WiFi fingerprint positioning and PDR positioning well, and the correction of WiFi fingerprint positioning fixes the accumulative error of the PDR positioning.

**Figure 14:**
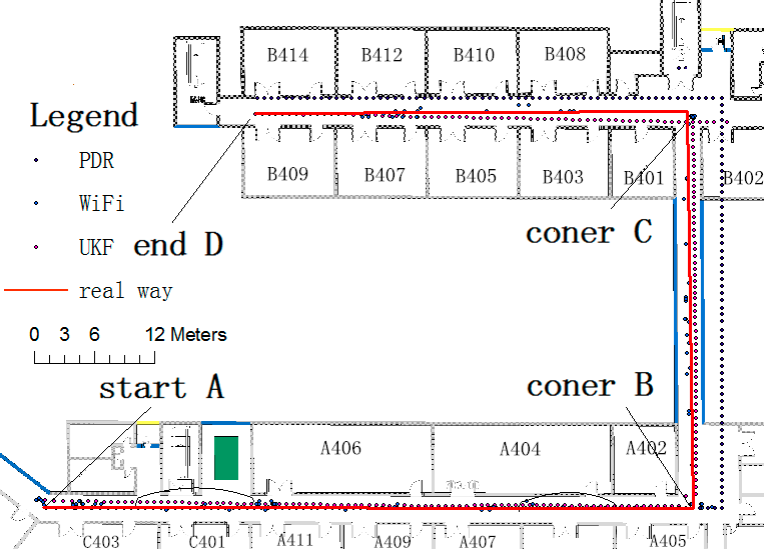
UKF positioning comparison test.

In order to test the effectiveness of the UKF fusion algorithm, the author made a contrast test from the opposite side in the original test site. The tester moved from the starting point A after two right-angle corners B and C, until to the end point D, for a total of 138.3 m, 219 steps. As shown in Figure 14, The UKF fusion method improved location results effectively compared to PDR positioning and WiFi positioning alone.

## 5. 3D Indoor Positioning System Design and Implementation

### 5.1. System Design

**Figure 15:**
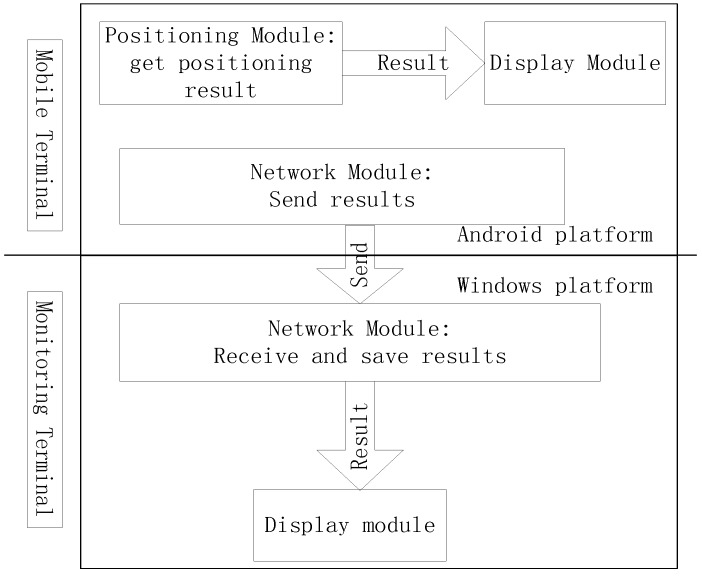
Structure of the 3D indoor positioning system.

The proposed 3D indoor positioning system is divided into three modules: positioning, display, and network. The positioning module contains the localization algorithm and obtains a person’s position. The display module includes the mobile and monitor modules. The mobile module displays the positioning result on the 3D scene on the mobile terminal and the monitor module displays all users’ positioning results in the 3D scene at the monitoring terminal. The network module is also further divided into mobile and monitor modules. The mobile module sends the user’s position information to the monitor module and the monitor module receives all online users’ positioning information and saves them into a database. The structure of the system is shown in Figure 15.

### 5.2. System Implementation and Test

**Figure 16:**
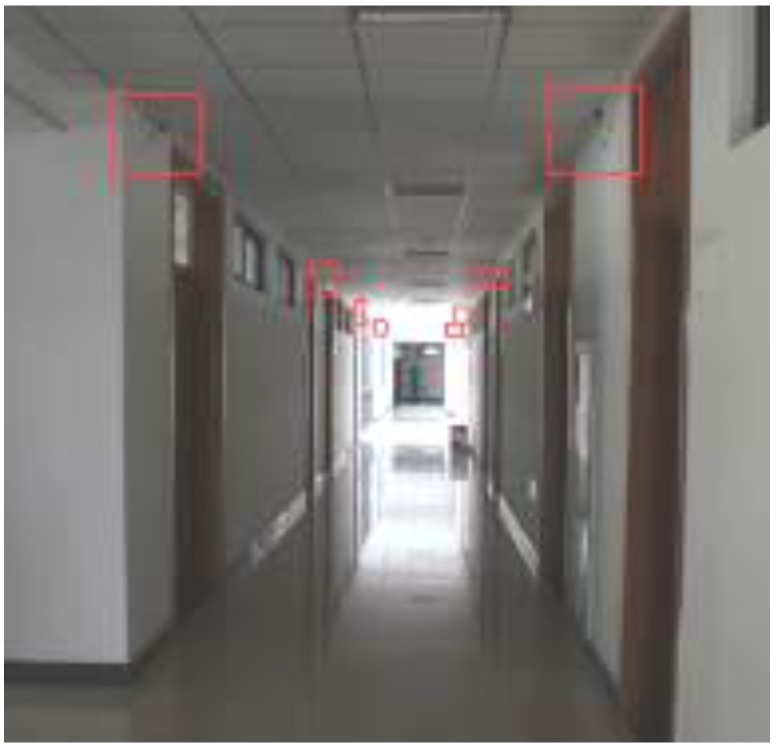
Indoor positioning test field.

**Figure 17:**
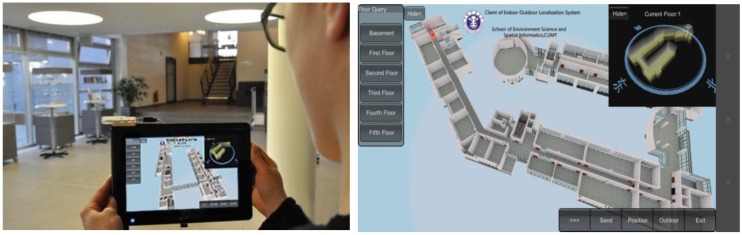
Interface of 3D-indoor Positioning System.

The implementation of 3D Indoor Positioning System is based on Unity 3D platform which can generate the real-time 3D scene in the mobile terminal. The Samsung Galaxy III (Android 4), equipped with a WiFi module and 1433 MHz CPU, was used as the mobile terminal test platform. The monitor terminal platform was a Lenovo G480 notebook computer (Lenovo, Beijing, China) whose operating system was Windows 7 Home Basic 64 bit, and was equipped with an Intel Core i5 2.5 GHz CPU. Our test field was in the School of Environment Science and Spatial Informatics, as mentioned above, and the real scene of the fourth floor corridor test area and the 3D scene in the positioning system are shown respectively in Figure 16 and Figure 17.

In the mobile positioning terminal, users cannot only acquire their location within the whole building as a bird’s eye view, but also switch to the current position indicated by the red point in the 3D scene, which changes to follow a user’s path. Meanwhile, users can acquire each floor of the whole building and send position information to the monitoring terminal. The monitoring terminal is responsible for showing all online users’ positions in the whole building and monitoring each floor. While tracking specified targets, it can switch 3D scenes to the appropriate monitoring floor and show the related information.

## 6. Conclusions

In this work, we proposed an integration of WiFi fingerprinting positioning and PDR that uses a UKF algorithm to improve the positioning accuracy and reliability. For WiFi fingerprinting localization, the improved K-means clustering algorithm was proposed to reduce the resource cost of the location algorithm and improve the system’s real-time performance without reducing the positioning accuracy. The time consumed was reduced on average by 51%, with the largest decline reaching 64% and the smallest value 36%. In the PDR approach, an auto-correlation analysis algorithm, which can greatly reduce the influence of mobile phone’s position and pedestrian’s motions on the result of step counting, was applied to count steps. To display the geographic information vividly, a 3D indoor positioning system based on the Unity 3D platform was devised. It can express complicated indoor space geographic information and enrich the user experience.
